# Heavy Metal Ions(II) Sorption by a Cellulose-Based Sorbent Containing Sulfogroups

**DOI:** 10.3390/polym15214212

**Published:** 2023-10-24

**Authors:** Tatiana Nikiforova, Vladimir Kozlov, Pavel Razgovorov, Natalia Politaeva, Ksenia Velmozhina, Polina Shinkevich, Valentina Chelysheva

**Affiliations:** 1 Department of Food Technology and Biotechnology, Ivanovo State University of Chemistry and Technology, Sheremetievskiy Avenue, 7, Ivanovo 153000, Russia; tatianaenik@mail.ru; 2 Department of Chemistry and Technology of Higher Molecular Compounds, Ivanovo State University of Chemistry and Technology, Sheremetievskiy Avenue, 7, Ivanovo 153000, Russia; kozlov@isuct.ru; 3 Institute of Civil and Transport Engineers, Yaroslavl State Technical University, Moskovsky Prosp., 88, Yaroslavl 150023, Russia; razgovorovpb@ystu.ru; 4 Institute of Civil Engineering, Peter the Great St. Petersburg Polytechnic University, Saint-Petersburg 195251, Russia; anizhomlev@mail.ru (K.V.); shinkevich.ps@edu.spbstu.ru (P.S.); chelysheva.valya@yandex.ru (V.C.)

**Keywords:** cellulose sorbents, sulfogroups, heavy metal ions, modification, aqueous solutions

## Abstract

This article concerns the effect of the chemical modification of short flax fiber on its sorption properties for heavy metal ions. The main purpose of the modification was to achieve the oxidation of flax cellulose with sodium metaperiodate to form dialdehyde cellulose. Additionally, the research shows the subsequent interaction of dialdehyde cellulose with 1-amino-8-hydroxynaphthalene-3,6-disulfonic acid and its transformation into a derivative capable of forming chelate complexes with heavy metal ions. Additionally, this article presents the results of equilibrium and kinetics studies of the sorption of Cu(II), Cd(II), and Fe(II) ions from aqueous solutions by primary and modified cellulose sorbents. SEM spectra indicate changes in the surface structure of the modified sorbents compared to the original one. IR spectra show the appearance of amino- and sulfogroups in short flax fibers in the process of their modification. The research revealed the efficiency of the method and the possibility of its use for the purification of aqueous solutions from heavy metal ions in industrial processes.

## 1. Introduction

Providing industrial enterprises and various consumers with water of proper quality is one of the most important tasks in emergency situations. Water supplies can be susceptible to many types of pollution. In order to achieve the required standards of water quality, we generally use water treatments. As a result, the composition of the water changes due to the use of various physicochemical methods of purification. The adsorption method is an environmentally friendly method of water purification. The essence of the method is the absorption of a substance from the environment by a solid, porous adsorbent.

Water purification is provided by natural [1,2,3,4] and synthetic [5,6,7] sorbents. When operating ion exchange plants for water softening and desalination, usually organic (artificial) synthetic ion exchangers are preferred. However, they incur relatively high costs. Therefore, nowadays more and more attention is being paid to the development of new inexpensive and effective ion exchangers. The cellulose-containing sorbents occupy a special place in this particular process [8,9,10]. Analysis of the physicochemical properties of these adsorbents allows a deeper understanding of the essence of the adsorption process as well as an assessment of their effectiveness and suitability for practical use.

Polysaccharide materials draw attention due to their environmental properties, low cost, hydrophilicity, ability to swell in aqueous solutions, and ability to bind various compounds. Additionally, their biocompatibility, biodegradability, mechanical strength, regeneration ability, recyclability, and abundance worldwide are taken into account.

The agricultural biomass wastes are cellulose, lignin, hemicellulose, proteins, extractives, waxes, oils, and starches containing active functional groups such as alcoholic, phenolic, carboxyl, carbonyl, and ester groups [11,12].

Cellulosic materials isolated from agricultural biomasses, including lignocellulosic plants, spent grain, rice husks, sawdust, wheat straw, tea waste, chitosan, sugarcane pulp, fruit waste, and weeds, are perspective sorbents for sorbing heavy metal ions from aquatic medium [13,14,15]. Numerous biomasses of agricultural waste can be successfully used to extract heavy metal ions from mono- and multicomponent wastewater [16].

The cellulose-based materials used for heavy metal ion sorption come from various raw plant materials, such as sawdust, tree bark, husks, flax fibers, the shells of nuts, seed grains, plant footstalks, herbaceous and aquatic plants, straw, silt, turf, seaweed, yeast, fungal biomasses, and bacterial biomasses [14,17,18,19]. Different waste products, such as fruit cortex waste [20], brewing industry spent grain [21], and others, are considered for use in the purification of water solutions from heavy metals.

Several studies have been performed [17,18,19] in the field of wastewater treatment using flax fibers. The choice of this plant material is due to its low cost and high availability. Flax fiber is widely used in the production of woven fabrics, insulation materials, and non-woven geotextiles. The results of these studies showed the good ability of flax for the sorption of zinc, copper, and lead ions under competitive and non-competitive conditions. The adsorption of the metal ions onto flax fibers [17] demonstrated maximum adsorption capacity values of 9.9, 10.7, and 8.4 mg/g, respectively, for Cu^2+^, Pb^2+^, and Zn^2+^. One study [18] demonstrated the adsorption of zinc, lead, and copper from contaminated wastewater treated with a flax biosorbent. The calculated maximum adsorption capacity values, 8.32, 13.35, and 7.12 mg/g, were obtained for Cu^2+^, Pb^2+^, and Zn^2+^, respectively. Competitive and non-competitive batch experiments were conducted on flax fibers to study the heavy metal ion biosorption performance [19]. The results under competitive conditions were different from those obtained in the non-competitive form. The maximum adsorption capacity values were 112, 122, and 71 for Pb^2+^, Cu^2+^, and Zn^2+^ in the single metal ion solution and 82, 57, and 8 mmol.kg^−1^ in the ternary metal ion solution, respectively, showing a high competition level between metal ions when added simultaneously. It should be noted that the biosorption efficiency of the unmodified sorbent was poor compared to commercial ion exchange resins.

Nowadays, bioadsorbents are widespread in medicine, cosmetics, the food industry, and electronic devices for the immobilization of enzymes, water purification, and other uses [22,23,24,25]. Recently, scientists have intensively developed environmentally friendly cellulose-based adsorbents for heavy metal sorption. The main problem is the absence of functional groups in cellulose capable of complex formation with heavy metals. Therefore, the sorption characteristics of cellulosic materials are not high enough. To solve this problem, cellulose is usually modified using different techniques. To develop new sorbents with improved sorption properties for the extraction of heavy metal ions, it is very important to choose an appropriate method for processing polysaccharide-based materials. Many methods, such as mechanical, physical, physicochemical, chemical, and biochemical approaches, have been proposed by various authors [11,26,27,28,29] to modify cellulose-based polymers, including flax fibers [27,29]. A promising method for modifying cellulose-containing sorbents is chemical modification, which can be conducted with inexpensive and accessible reagents, such as organic and mineral acids, bases, organic compounds, and oxidants. Indeed, all of them can be used to modify cellulose-based polymers.

The goals of modification are to increase the availability of sorption-active groups, as well as to increase the internal adsorption surface and create new sorption centers enhancing the sorption capacity of natural sorbents [14].

Cellulose-based sorbents can be functionalized by esterification [30], etherification [31], sulfonation [32], and oxidation [33]. It makes them more qualitative and efficient adsorbents. The interest of researchers in the materials of cellulosic nature as adsorbents is increasing significantly due to their highly efficient adsorption of heavy metal ions [34,35].

These biopolymers can also be used for sorption of various heavy metal ions in grafted form [11]. They can also act as fillers, matrices, and coatings/shells; additionally, they can be in the form of gels, fibers, films, beads, membranes, etc. [36,37].

The purpose of this research is to obtain a new, effective sorbent containing sulfonic acid groups (-SO_3_H) by chemical modification of flax fiber and determine its advantages over sorbents containing -COOH groups for sorption of heavy metal ions from aqueous acid solutions.

## 2. Materials and Methods

### 2.1. Chemicals

Metal salts such as CuSO_4_·5H_2_O, FeSO_4_·7H_2_O, and CdSO_4_·8/3 H_2_O were used as the metal ions sources; sodium metaperiodate NaIO_4_ and 1-amino-8-hydroxynaphtalene-3,6-disulfoacid (C_10_H_9_NO_7_S_2_, H acid) were used as reagents for sorbent modifying. All used reagents were chemically pure.

We studied a secondary product of flax industry processing—short flax fiber, with the composition, wt%: cellulose 75–78; hemicellulose 9.4–11.9; lignin, 3.8; pectins 2.9–3.2; waxy substances 2.7; nitrogen-containing substances calculated for proteins 1.9–2.1; and mineral substances 1.3–2.8 [27].

### 2.2. Modification of Flax Fiber

Short flax fiber was used as a sorbent for modification; it was boiled with 5% solution of NaHCO_3_ for 30 min in order to remove impurities and improve its hydrophilicity and porosity. After boiling, it was washed with distilled water and dried to constant weight. The dried samples had an 8% moisture content.

The following conditions were chosen for the oxidation of flax cellulose: a concentration of sodium metaperiodate solution of 0.1 mol/L, pH 4, a temperature of 40 °C, a modulus oxidizer solution/flax cellulose of 100, and an oxidation time of 48 h.

A sample of flax fiber was put in a flask with a lapped stopper containing 0.1 N solution of sodium metaperiodate with a module solution/sorbent M = 50. The flask contents were stirred at room temperature in the dark using a magnetic stirrer. During the oxidation process, the concentrations in the solution of IO_4_^−^ ions were determined in the samples taken at certain intervals. The optical density of these solutions at a wavelength of 225 nm was measured using Spectrophotometer U-2001. The process was completed when the optical density of NaIO_4_ solutions stopped decreasing.

At the end of the periodic oxidation reaction, the oxidized sorbent was filtered off from the reaction products solution and sequentially washed with 1 L of dilute water solution of hydrochloric acid (pH 1); then, it was washed with 1 L of acetone/water 1/10 mixture (control with solution of silver nitrate); and finally, it was washed with 75–100 mL of acetone and dried. The aldehyde group content in flax fiber before and after oxidation was detected by iodometric titration [38,39].

The dialdehyde cellulose was then treated with HAD acid. The reaction was carried out at a molar ratio of 1:1 in an aqueous solution with a solution/sorbent module 20 for 1 h at pH 11–12 and room temperature.

### 2.3. Batch Experiments

Sorption kinetics and sorption equilibrium were investigated according to [40]. The kinetics of sorption was investigated under static conditions with stirring by the method of limited solution volume. To obtain kinetic curves of sorption, weighed portions of (*m*) sorbent of 0.1 g each were filled with 10 mL (*V*) of an aqueous solution of metal sulfate and kept for 5 min to 24 h with stirring at a temperature of 273 K. Initial concentration *C*_0_ of metal ions was 1.5 × 10^–4^ mol/L. At certain time intervals, the solution was separated from the sorbent by filtration, with the current concentration of metal ions C_τ_ being found by atomic absorption spectroscopy on a 210 VGP device. Sorption capacity *q_τ_* of sorbents at each given moment was calculated by the following formula:

$$q_{\tau }=\frac{\left(C_{0}-C_{\tau }\right)}{m}\cdot V$$

To obtain sorption isotherms, weighed portions (*m*) of sorbent of 0.1 g each were filled with 10 mL (*V*) of an aqueous solution of copper sulfate with initial concentrations C_0_ of 5 × 10^–4^–5 × 10^–2^ mol/L and kept until equilibrium was reached at a temperature of 273 K. The solution was then separated from the sorbent by filtration, and the equilibrium concentration of metal ion *C* was determined by atomic absorption spectroscopy on a 210 VGP device.

Under the conditions of steady equilibrium in the system, the equilibrium sorption capacity of the sorbents *A* was found:

$$A=\frac{\left(C_{0}-C\right)}{m}\cdot V$$

The degree of extraction *α* was determined as follows:

$$\alpha =\frac{C_{0}-C}{C_{0}}\cdot 100\%$$

In calculating the relative bias of experiments, each point was defined as the values taken from three parallel experiments. The error of «210 VGP» apparatus in determining the concentration of metal ions was 3%, and the error of the experiment was about 10%. Biological testing of water quality was based on the use of Daphnia magna Straus as a test object in relation to aqueous extracts from samples of short flax fiber modified according to the proposed method.

## 3. Theory

To improve the sorption capacity of the sorbents, various groups can be introduced in their structure. Therefore, -COOH groups are often used. Carboxyl groups can sorb heavy metal ions by ion exchange or complexation with -OH, -COO, or other neighboring groups. However, the dissociation constants of such sorption centers (pKa -COOH ≈ 4–5) [41] are quite low. It accounts for the high sensitivity of such sorption centers to an increase in the acidity of the medium. COOH- groups at low pH transfer to the undissociated state and lose their activity.

With increasing acidity (at pH < 3), the process of proton desorption starts. It transforms COO-sorption centers from an anionic active form to an H-acidic one, which is practically inactive for sorption of metal cations from acidic aqueous media. It can be expressed by reactions without taking into account the hydration of ions in phases:

− Sorption of M^2+^:

{Cell(-COO^–^)_2_ + M^2+^ ↔ Cell(-COO)_2_M}; (1)

− Activation–deactivation of sorption centers:

{Cell(-COOH)_2_ ↔ Cell(-COO^–^)_2_} + 2H^+^; (2)

− Competitive exchange (sorption of M^2+^ and proton desorption of M^2+^)

M^2+^ + {Cell(-COOH)_2_ ↔ Cell(-COO^−^)_2_M^2+^} + 2H^+^. (3)

Therefore, to increase the sorption activity of biosorbents during the process of sorption of M^2+^, it is necessary to consider the number of acid sorption centers and the effect of acidity of the aqueous phase on their activity. Strong sulfonic acids with sufficiently large dissociation constants are suitable for these purposes. Usually, sulfonic acids form rather stable monohydrates in the form of hydroxonium arenesulfonates ArSO_3_^−^H_3_O^+^ [41].

In this case, the sorption process in a wide pH range as a competitive mechanism of ion exchange sorption process and proton desorption with the participation of metal cations M^2+^, hydroxonium ions H_3_O^+^, and sulfonic groups of the sorbent will proceed as follows:

M^2+^ + {Cell(-SO_3_^−^H_3_O^+^)_2_ ↔ Cell(-SO_3_^−^)_2_M^2+^} + 2H_3_O^+^. (4)

All characteristics of the interphase ion exchange mechanisms of metal cation to metal cation (M^2+^/2M^+^—ion exchange) and metal cation to proton (M^2+^/2(H^+^aqA^−^)—proton desorption) are determined exclusively by the different states of metal cations and proton in the aqueous phase.

Sorption of heavy metal ions by modified biopolymer sorbents from aqueous solutions of strong acids depends on the state of metal cations and protons in the phases, the dissociation mechanism of weakly acidic (-COOH) and strongly acidic (-SO_3_H) sorption centers, and a significant difference in the hydrate-proton activity of dilute acid solutions in the pH range from 7 to 0 and over its range in the H_0_ range from 0 to −5.5.

Weak acids dissociate with the release of one “free” proton, and their dissociation constants can be expressed as the value of the solution acidity on the pH scale at the point of semi-conversion of conjugated forms of bases.

R-COOH ↔ R-COO^−^ + H^+^; (5)

pK_aRCOOH_ = pH + log(I) = pH when I = (RCOOH/RCOO^−^) = 1. (6)

Strong acids dissociate in the acidity area out of the pH scale with the superposition of hydration processes involving two “free” protons with different activities. This is due to the different forms of participation of excess acidity X = (fH + fB/fBH) and dissociation constants expressed on the Ho scale as the half-conversion points of the two hydrated conjugated forms of the substrate pKa Ar-SO_3_H = (1/2) (pKa_1_ + pKa_2_):

Strong acids dissociate in the acidity area out of the pH scale with overlapping sequentially occurring processes of hydration of conjugated forms of the substrate with the participation of two “free” protons with different activities,

Ar-SO_3_H·H_3_O^+^ ↔ ↓Ar-SO_3_^−^·H_3_O^+^ + H^+^ (pK_a1_), (7)

↓Ar-SO_3_^−^·H_3_O^+^ ↔ Ar-SO_3_^−^·H_2_O + H^+^ (pK_a2_). (8)

In the area of excess, basicity heavy metal cations form poorly soluble hydroxides and metal oxides, which precipitate or co-precipitate on the surface of the solid phase.

In the area of excess acidity (pH ≤ 7) sorption of heavy metal cations from aqueous solutions of salts on biosorbents having acidic sorption sites proceeds by the mechanism of ion exchange of M^2+^ to 2 (M^+^) due to their practically identical state in electroneutral phases.

Ion exchange:

(2A^−^) + M^2+^ + {Cell(-COOM)_2_ ↔ Cell(-COO)_2_M} + 2M^+^ + (2A^−^). (9)

Proton desorption:

n (H_2_O) + (2A^−^) + M^2+^ + {S(-COOH)_2_ ↔ S(-COO)_2_M} + 2{[H (H_2_O)_n_] + A^−^}. (10)

It is important to consider the significant differences in the mechanisms of the weak (-COOH) and strong (-SO_3_H) acid groups’ dissociation.

## 4. Results

### 4.1. Chemical Modification of a Sorbent

Cellulose-containing sorbents, as a rule, have a relatively low sorption capacity and moderate kinetic characteristics. Therefore, to increase their efficiency, it is necessary to modify the structure of the cellulose-containing material using the available reagents and simple technological operations if possible. To improve the kinetic and equilibrium properties of flax fiber, it is important to generate sorption-active groups on its surface. Chemical modification was conducted in two stages:

1. Flax cellulose oxidation was performed with NaIO_4_ to form aldehyde groups on cellulose;
2. Dialedhydocellulose was treated with H-acid to obtain the modified cellulose.

To achieve maximum results in modifying flax cellulose, the optimal conditions in each of these stages were revealed.

Cellulose oxidation can be performed by using oxidants such as HIO_4_, NaIO_4_, NaOCl, NaClO_2_, H_2_O_2_, O_3_, K_2_Cr_2_O_7_, KMnO_4_, and sodium chlorite.

The action type of most of these oxidants is nonspecific, and their application results mainly in the formation of -COOH groups as well as in the breaking the macromolecule cellulose chain.

When cellulose is treated with periodic acid and its salts in aqueous solutions, the formation of 2,3-dialdehyde cellulose occurs [42]. The oxidation of cellulose by sodium metaperiodate results in the oxidation of two neighboring OH- groups at the C_2_ and C_3_ up to aldehyde groups with the simultaneous–C-C– bond rupture and formation of dialdehyde cellulose without primary alcohol group oxidation [42]. The periodate ions interact with cellulose without the destruction of fibers. 

**Scheme 1:**
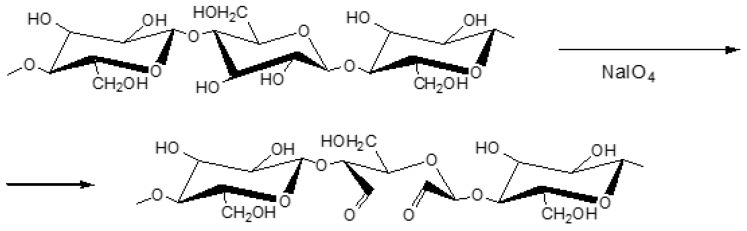
Selective cellulose oxidation with sodium metaperiodate.

The oxidation of flax cellulose with sodium metaperiodate can be presented as follows (Scheme 1):

At the initial stage, the cellulose oxidation is largely confined to the readily accessible amorphous area. The oxidation of cellulose with NaIO_4_ under special conditions can result in products with a high content of carboxyl functional or acid endiol groups. To avoid radical-induced depolymerization reactions, it is necessary to conduct the reaction in the dark.

The choice of optimal oxidation conditions for cellulose was made by varying the oxidant concentration and the oxidation time. The important parameters affecting this process are the solution pH level, the temperature, and the solution/adsorbent ratio. Oxidation of flax cellulose was performed in acidic solutions (pH 3–4), in which the reactivity of NaIO_4_ was high.

Therefore, the most preferred conditions are the following ones: concentration of sodium metaperiodate solution—0.1 mol L^−1^, 40 °C, pH 4, solution/cellulose ratio—100, oxidation time: 8 h [37,38].

The dialdehyde cellulose obtained by flax cellulose oxidation with a solution of NaIO_4_ contained 12% aldehyde groups (the aldehyde group content in natural flax fiber was 0.4%). Then, dialdehyde cellulose was treated with 1-amino-8-hydroxynaphtalene-3,6-disulfonic acid.

Taking into account the variety of existing methods, the chemical modification of cellulose-containing sorbents seems to be a promising one. This makes it possible to increase the adsorption capacity of the natural sorbents in terms of using the available and inexpensive reagents.

For this purpose, to obtain a new sorbent containing -SO_3_H groups, we studied the process of chemical modification of flax cellulose. On the one hand, it is non-hazardous (biologically inert and environmentally friendly) to the treated media; on the other hand, it demonstrates high sorption characteristics in aqueous solutions and is capable of strong binding to heavy metal ions.

Aldehyde groups of the dialdehyde cellulose macromolecules in positions C_2_ and C_3_ allow us to obtain a derivative able to form complexes of chelate type with metal cations.

**Scheme 2:**
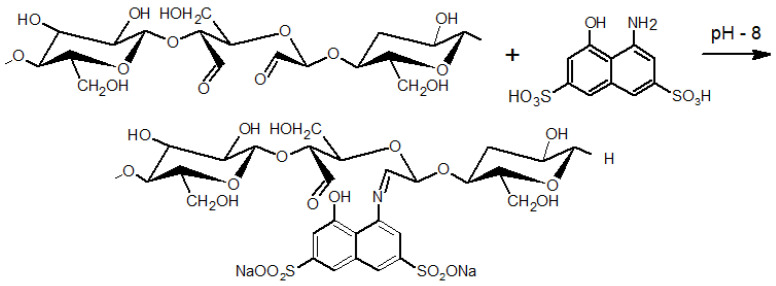
Modification of dialdehyde cellulose with H-acid.

The modification of peroxidized flax fibers was conducted in an aqueous H-acid solution at a 1:1 molar ratio and solution/sorbent ratio 20 for 1 h at pH 7–10 and room temperature. Flax fiber dialdehyde cellulose reacts with amino groups of H-acid to form a Schiff base. As a result of the modification, the sulfogroups attached to the aldehyde groups of the cellulose units (Scheme 2).

The purification of the modified fiber from unreacted reagents was conducted by washing it with distilled water to achieve a neutral reaction. Then, the sorbent was dried to a constant weight, and its sorption properties were studied.

### 4.2. Sorption Properties of Natural and Modified Flax Fiber

**Figure 1:**
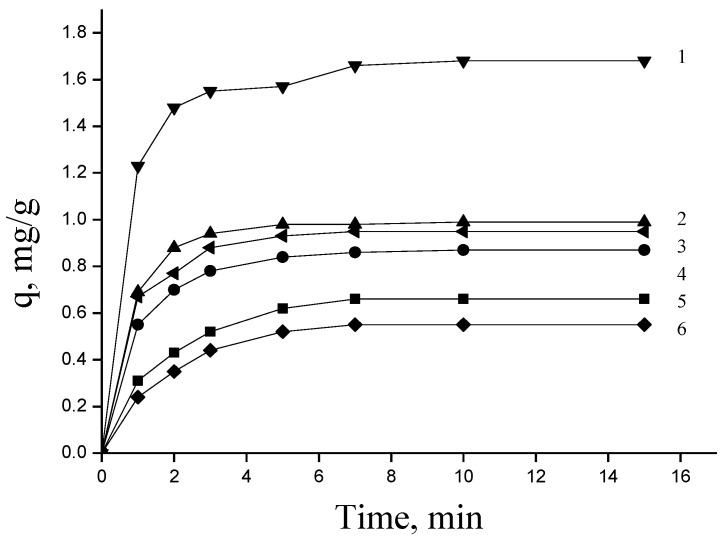
Sorption kinetics of Cd^2+^ (1, 2), Cu^2+^ (3, 5), and Fe^2+^ (4, 6) ions on natural (2, 5, 6) and modified (1, 3, 4) flax fiber from aqueous solutions: ▼—Cd^2+^, ▲—Cd^2+^, 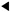
—Cu^2+^, ●—Fe^2+^, ■—Cu^2+^, ♦—Fe^2+^.

The sorption ability of modified flax fiber to heavy metal ions was studied. To determine the time of reaching the sorption equilibrium in the system “aqueous solution—sorbent”, kinetic curves of sorption of Cd^2+^, Cu^2+^, and Fe^2+^ ions from aqueous solutions of their sulfates by samples of natural and modified flax fiber were obtained. The experimental results are presented in Figure 1.

As the kinetic studies showed, the time to reach sorption equilibrium in the heterophase system “aqueous solution of metal sulfate—sorbent” for modified sorbents was slightly reduced compared to natural flax. At the same time, the sorption of heavy metal ions by modified sorbents increased.

The adsorption data obtained were processed using Lagergren’s “pseudo first-order model” and Ho’s “pseudo second-order model:

(11) $$\mathrm{pseudo}\;\mathrm{first}-\mathrm{order}\;\mathrm{kinetics}:\;q_{t}\;\;=\;\;q_{eq}\;(1\;\;-\;\;e^{-k_{1}t});$$

(12) $$\mathrm{pseudo}\;\sec\mathrm{ond}-\mathrm{order}\;\mathrm{kinetics}:\;q_{t}\;\;=\;\;\frac{t}{\frac{1}{k_{2}*q_{eq}^{2}}\;\;+\;\;\frac{1}{q_{eq}}}.$$

where *k*_1_ (min^−1^) and *k*_2_ (mg min g^−1^) are the pseudo first- and pseudo second-order rate constants of adsorption, respectively; *q_t_* (mg g^−1^) is the adsorption capacity at time *t*; *q_e_* (mg/g) is adsorption capacity at equilibrium.

The processing of kinetic curves of sorption of heavy metal ions by native natural and modified flax fibers in terms of pseudo first- and pseudo second-order kinetic models is shown in (Supplementary Material S1). All the kinetic parameters received from the intercepts and the slopes of respective plots are presented in Table 1 (293 K; *C*_0_ = 1.5 × 10^–4^ mol L^–1^).

The data presented in Table 1 indicate that the pseudo second-order model describes the adsorption kinetics obtained in this work more correctly (*R*^2^ = 0.99 and *q_e_* ≈ *q_e_* experimental). 

The next step was to determine the diffusion effect of Cd^2+^, Cu^2+^, and Fe^2+^ ions in the adsorbent pore structure on the sorption kinetics. Usually, for this purpose, an intraparticle diffusion model based on the theory proposed in [43] is used in the form of

(13) $$q_{t}\;\;=\;\;k_{id}\;t^{1/2}+C_{i},$$

where *k_id_* (mg/(g min^1/2^)) is the intraparticle diffusion rate constant, and *C_i_* (mg/g) is the intercept of step i representing the boundary layer thickness.

The plots of *q_t_* versus *t*^1/2^ are shown in (Supplementary Material S2).

Many studies report good correspondence with the PSO model as well as models based on diffusion as the limiting stage.

The studies reported in [4], as well as in Table 1, showed very high coefficients of determination for the PSO model as well as good agreement with the diffusion-based model proposed by Weber and Morris.

### 4.3. Equilibrium Characteristics of Sorption of Cu^2+^, Cd^2+^, and Fe^2+^ Ions by Natural and Modified Flax

**Figure 2:**
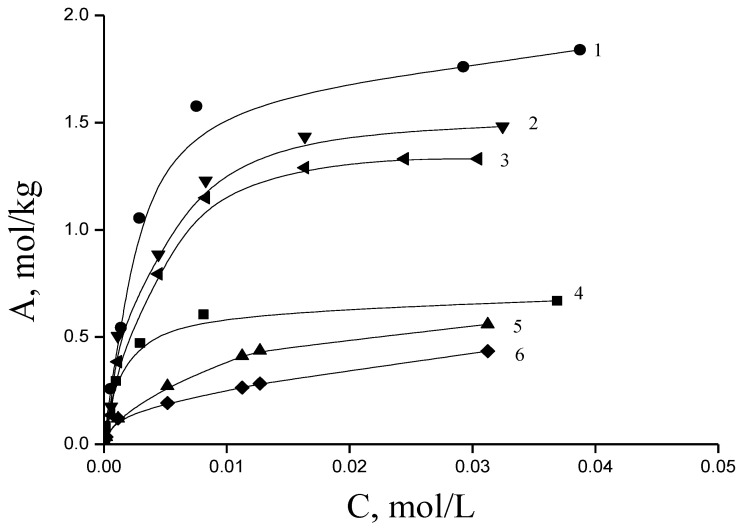
Sorption isotherms of Cu^2+^ (1, 4), Cd^2+^ (2, 5), and Fe^2+^ (3, 6) ions from aqueous solutions on natural (4, 5, 6) and modified (1, 2, 3) flax fiber.

To investigate the equilibrium of Cd (II), Cu (II), and Fe (II) ion sorption by natural and modified flax fibers from aqueous solutions of their sulfates, the isotherms of sorption of heavy metal ions were received (Figure 2). The Langmuir and Freundlich adsorption models were used to describe the process of heavy metal ion sorption on flax fibers (Supplementary Material S3).

The experimental sorption isotherms are well described by Langmuir adsorption isotherm model.

(14) $$A\;\;=\;\;\frac{{A_{\infty }\cdot K\cdot C}_{e}}{(1+K\cdot C_{e})}\;,$$

where *K_L_* is the concentration constant of sorption equilibrium characterizing the intensity of the sorption process, L mol^–1^; *A* is the adsorption capacity at equilibrium; *A*_∞_ is the maximum (limiting) sorption capacity of the sorbent for a given metal, mol kg^–1^; and C is the equilibrium concentration of a heavy metal ion in a solution, mol L^−1^.

The absence of an intersection and divergent character of isotherms indicates that the sorption of heavy metal ions occurs in the same sorption centers with their filling in accordance with the exponential law.

The experimental isotherms of sorption were processed in linear coordinates of the Langmuir equation:

(15) $$\frac{C_{e}}{A}\;\;=\;\;\frac{C_{e}}{A_{\infty }}\;\;+\;\;\frac{1}{A_{\infty }\cdot K}\;.$$

The treatment of the sorption isotherms of Cu^2+^, Cd^2+^, and Fe^2+^ ions from aqueous solutions in the linear form of the Langmuir equation by the least squares method yields the values of the maximum (limiting) sorption *A*_∞_ and the *K_L_* constant (Supplementary Material S3).

These constants for the sorption of Cd^2+^, Cu^2+^, and Fe^2+^ ions on natural and modified sorbents and the correlation coefficient R are provided in Table 2.

The Freundlich isotherm model is usually used in the form of the linearized equation:

(16) $$\ln q_{e}\;\;=\;\;\ln K_{F}\;\;+\;\;\frac{1}{n}\ln C_{e}\;,$$

where *K_F_* (mg g^−1^)(L mg^−1^)^1/n^ and 1/n are empirical constants indicating the adsorption favorability. The *K_F_*, *n*, and *R* obtained by fitting the experimental data with the Freundlich equation (Supplementary Material S3) are presented in Table 2. Thus, a value of n in the range from 1 to 10 indicates favorable adsorption. For the selected experimental conditions, the data obtained are well described by both the Langmuir isotherm (R = 0.99) and the Freundlich isotherm (0.96–0.98).

Table 2 shows that the limiting sorption of flax fiber as a result of modification increases by 2.9 times for Cu^2+^ and Fe^2+^ ions and by 2.4 times for Cd^2+^ ions. The obtained *A*_∞_ values indicate the higher sorption properties of the modified sample in relation to metal ions, which are comparable with those of cation exchange resin Lewatit S100.

According to the experimental results, the *A*_∞_ of flax fiber modified with H-acid in relation to heavy metal cations increases as follows:

Fe (II) < Cd (II) < Cu (II).

The maximum sorption capacity of the modified sorbent based on flax fiber toward the heavy metal ions is superior to many sorbents of a cellulose nature, the capacity of which covers the range of 0.5–1 mol/kg as a rule [14,17,18]. A comparison of the maximum adsorption capacities of the different low-cost adsorbents on the base of flax is presented in Table 3.

The observed values of Langmuir constants (*K*) and maximum sorption capacities (*A*_∞_) for cations of various heavy metals show the differences in their coordination on the sorption sites of sorbents on the base of cellulose and also in their binding strength in the phases, as well as in their hydration in the aqueous phase.

The sorbent was regenerated with 1 N sodium chloride solution for 1 h, and then the sorbent was washed with distilled water and dried to a constant weight. It was found that after seven cycles of “sorption-desorption”, the sorption capacity of the sorbent decreased by 43% compared to the initial capacity.

### 4.4. The Effect of pH on the Sorption of Cu^2+^ Ions by Modified Flax Fiber

To control the process of chemisorption of d-metal cations from acidic aqueous solutions by biosorbents, it is important to understand the effects of the acidity (pH) of the aqueous phase on the state of metal cations and protons in the aqueous solution and the sorbent.

The value of the equilibrium exchange capacity of the sorbents is determined by the solution acidity. It is associated with the influence of solution acidity on the processes of complexation, electrostatic interaction during physical adsorption, and the charge of the sorbent surface.

To determine the optimal range of acidity values, we study the effect of the pH of the solution on the sorption of Cu^2+^ ions by the modified flax fiber in aqueous solutions of metal sulfate. The pH range (pH > 7) is associated with the beginning of the precipitation of heavy metal hydroxides in the alkaline pH range.

**Figure 3:**
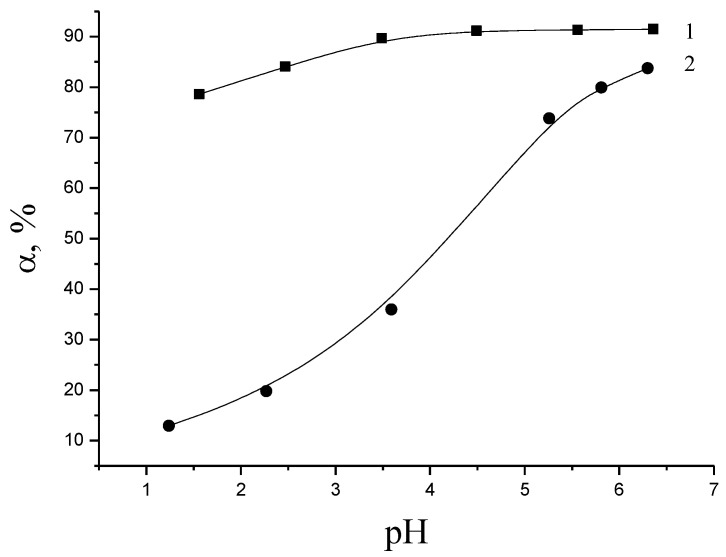
Effect of pH of equilibrium solution on sorption of Cu^2+^ ions.

Figure 3 (curve 1) shows that the sorption degree of copper ions by modified flax fiber is more than 90% and weakly depends on the acidity of the medium in the pH range of 1–7. Such behavior is typical for strongly acidic cation exchangers. A slight increase in sorption is characterized by deprotonation of sorption sites of the sorbent (-SO_3_H → -SO_3_^−^) with decreasing acidity of the medium.

Although the maximum extraction of Cu (II) cations by the modified polysaccharide sorbent is observed at the pH of equilibrium solutions close to neutral media, in contrast to weakly acidic cation exchangers containing carboxyl groups, the obtained sorbent can exhibit sorption properties in a wide pH range. It is characteristic of strongly acidic cation exchangers.

The study of their behavior (Cell-SO_3_H) during the extraction of heavy metal ions from aqueous–acidic media indicates their greater resistance to proton desorption in the acidic pH area compared to sorbents of the (Cell-COOH) type (Figure 3, curve 2).

### 4.5. IR Spectra

Flax fiber contains a mixture of polymers: cellulose, lignin, hemicellulose, pectin, and protein substances with various functional groups. As a result of oxidation and subsequent treatment with H-acid, changes occur in the structure of the flax. 

**Figure 4:**
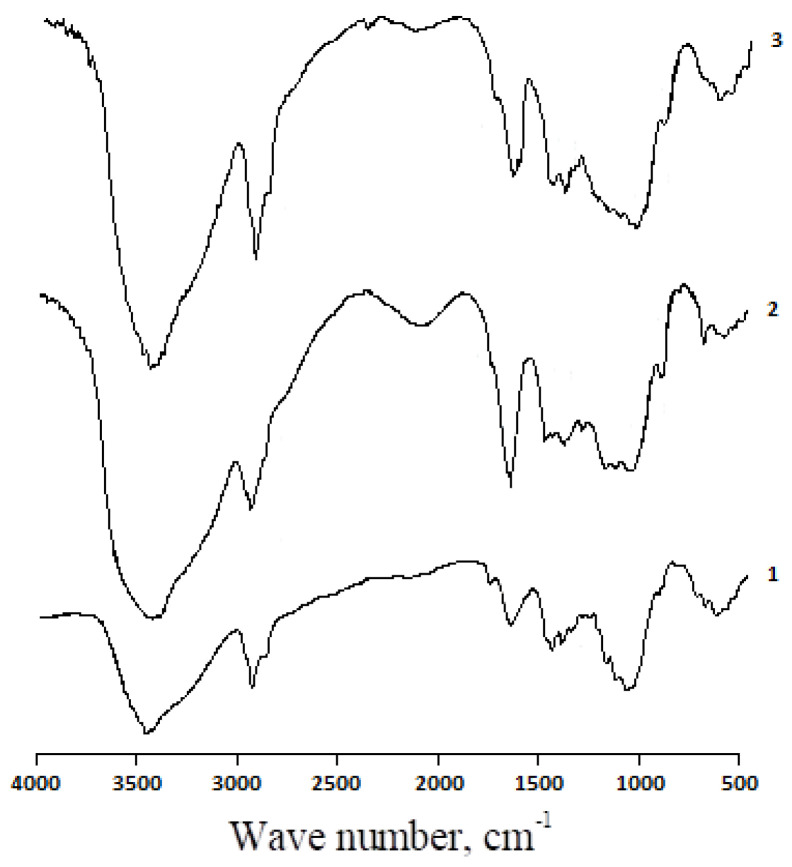
IR spectra of flax fibers: 1—natutal; 2—oxidized with NaIO_4_; 3—modified with AHD H-acid.

To confirm these changes, the IR spectra of the natural flax fibers and flax fibers oxidized with sodium metaperiodate and modified with H-acid were analyzed. The IR spectra of natural, oxidized, and modified flax fibers are shown in Figure 4.

The IR spectra of all the samples showed valence vibrational bands at 1740 cm^−1^. This is due to the presence of carboxyl groups in pectin compounds, cellulose, and hemicellulose.

As a result of the chemical modification of the natural sorbent sample, new functional groups in the structure of the fiber appeared.

Very significant changes in the spectra are observed in the range of 1640–1600 cm^−1^, which shows stretching vibrations of carbonyl groups in aldehydes and carboxylic acids.

The content of aldehyde groups in the natural fiber is small. Therefore, there is a small peak at 1634 cm^−1^, corresponding to CHO groups in its IR spectrum.

After oxidation with sodium metaperiodate, this peak increases significantly and shifts to 1638 cm^−1^ in the IR spectrum of oxidized fibers. It confirms the formation of aldehyde groups in flax fibers. In the sample modified with H-acid, a peak is observed at 1633 cm^−1^ and a new band appears at 1606 cm^−1^. This may be related to the appearance of amino groups in the modified sample.

In addition, the 1400–1300 cm^−1^ area exhibits symmetric stretching vibrations of the S=O group and strain vibrations of the C-H bond. The spectrum pattern changes in this part of the IR spectrum. In the modified sorbent, there are two distinct bands at 1458 cm^−1^ and 1381 cm^−1^. These regions are represented by multiple small peaks in the spectra of the original and oxidized flax.

In the area of 1650–1450 cm^−1^, the deformation vibrations of the N-H bond in amines are revealed. The detection of such bands in the spectrum of a flax fiber sample modified with H-acid indicates the fixation of acid on the sorbent.

Hence, the IR spectra indicate changes in the structure of flax fiber during various processing methods, for instance, the oxidation process under the action of sodium metaperiodate with the formation of dialdehyde cellulose and subsequent modification with H-acid with the formation of new functional sorption-active groups on the sorbent.

### 4.6. SEM Study of the Sorbent Structure

**Figure 5:**
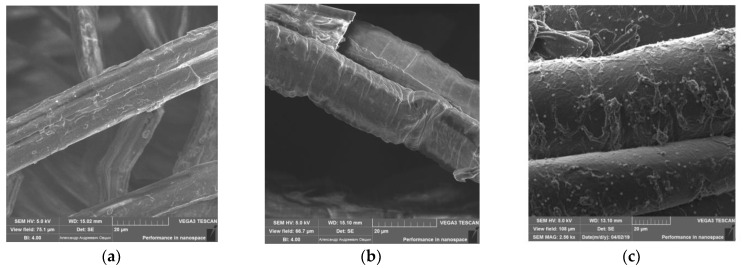
SEM images of the flax fiber samples surface layer: (**a**)—natural flax fibers; (**b**)—flax fibers oxidized with sodium metaperiodate; (**c**)—flax fibers modified with H-acid.

The study of the sorbent samples’ surface structure by electron microscopy showed the changes in the flax fibers’ surface layer microrelief during modification. Images of the natural, oxidized, and modified flax fibers obtained with a VEGA 3 SBH scanning electron microscope are shown in Figure 5a–c. The surface of the pristine flax (Figure 5a) is heterogeneous, with a large number of folds and inclusions since flax fibers are formed from elementary fibers bonded together by middle plates consisting of lignin and pectin substances. During oxidation with sodium metaperiodate (Figure 5b), smoothing of the fiber structure and removal of various inclusions from the surface are observed. In the process of flax modification with H-acid (Figure 5c), folds and creases appear on the surface of the fibers. Thus, these SEM images indicate changes in the surface of the samples under study. This indicates the occurrence of two various types of modifications to the flax fibers.

The change in the elemental composition of flax fiber in the process of its oxidation with sodium metaperiodate and modification with H-acid is shown in (Supplementary Material S4).

Consequently, the microscopic studies using the SEM method show the changes in the surface structure and elemental composition of the flax fiber under study during its oxidation with dialdehyde cellulose and modification with H-acid.

The sorbent modified with H-acid is characterized by increased sorption properties toward heavy metal ions. This is demonstrated with clear evidence by the elemental analysis of the surface of sorbent samples (pristine, oxidized, and modified ones) after the sorption of Cu^2+^ ions (Supplementary Material S4). The copper content in the samples under study increases in the following order: oxidized flax fibers (0.12%) < pristine flax fibers (1.78%) < modified flax fibers (10.66%).

These results are consistent with the data on heavy metal ion sorption by the natural, oxidized, and modified samples of flax fiber. Thus, the formation of aldehyde groups in the polymer chain of cellulosic materials does not form additional sorption sites for metal cations in such biosorbents. However, -CHO groups make it possible to introduce fragments containing sorption active groups (e.g., sulfogroups) through nucleophilic addition reactions and cleavage reactions.

This determined a method to modify cellulosic materials in order to obtain biosorbents with a high sorption capacity (Cell-SO_3_H) and higher resistance to proton desorption in an aqueous acidic medium than Cell-COOH sorbents.

### 4.7. Ion Exchange Purification of Solutions from Heavy Metal Ions in a Single-Chamber Apparatus with a Fluidized Bed

**Figure 6:**
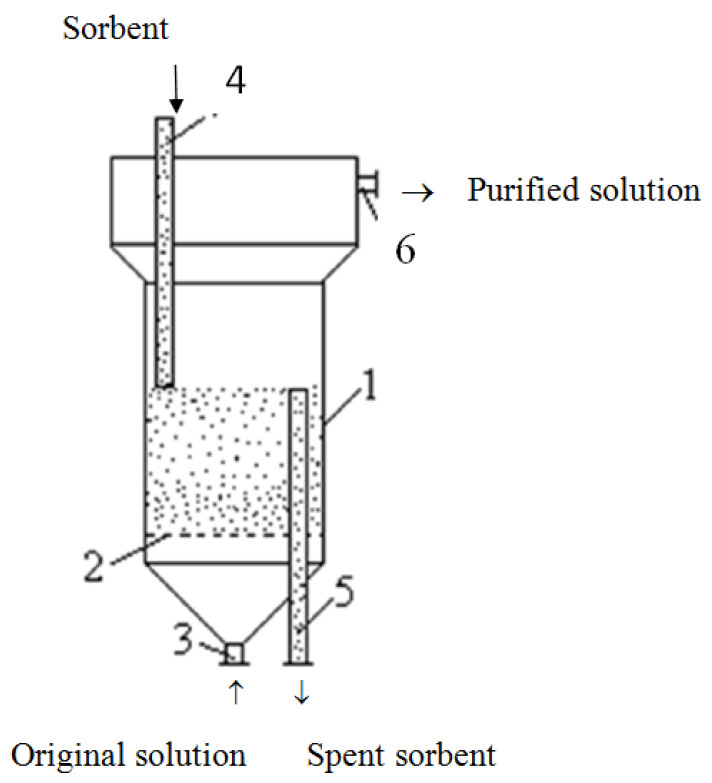
One-section device with fluid bed of ionite: 1—body; 2—distributed grid; 3, 4, 6—pipe junction; 5—tube.

In this research, we conducted the process of solutions for ion exchange purification from heavy metal ions in a single-chamber apparatus with a fluidized bed of a modified sorbent (Figure 6).

For the experiments, we used an ion exchange apparatus with a cylindrical body of 0.08 m in diameter and a conical bottom. In the lower part of the apparatus, there was a distribution grid 3 × 10^−3^ m thick with a hole diameter of 2 × 10^−3^ m and a flow section of 20.6%. The height of the fluidized bed of the sorbent in the apparatus was 0.12 m. In the upper part of the apparatus, there was a chamber for separating solid and liquid phases. It is a cylinder of 0.15 m in diameter and 0.1 m high, joined to the apparatus body by a conical shell.

The original solution was continuously supplied through pipe junction 3 to body 1, where it moved in the direction from bottom to top, maintaining the sorbent in a fluidized state on the distribution grid 2. Simultaneously, regenerated sorbent was fed into the apparatus through branch pipe 4, which, after saturation with metal ions, was removed through branch pipe 5. The height of the fluidized bed of the sorbent in the apparatus is determined by the height of tube 5 above the distribution grid 2. The purified solution was removed from the upper part of the apparatus through pipe junction 6.

We assessed the ion exchange in the system of “weakly concentrated MSO_4_ solution—modified sorbent”. The productivity of the apparatus for the solution was 2.1 × 10^−5^ m^3^ s^−1^ and for the sorbent, 1.42 × 10^−7^ m^3^ s^−1^. The concentration of solutions of heavy metal ions supplied for treatment was 5.1 × 10^−3^ kg-eq m^−3^. According to the experiment results, the concentration of metal ions in the cation exchanger at the outlet of the apparatus was 0.71, 0.67, and 0.65 kg-eq m^−3^ for copper (II), cadmium (II), and iron (II) ions, respectively.

### 4.8. Biological Testing of Water Quality with Daphnia Magna Straus

Biological testing of water quality was performed according to the method for determining the acute toxicity of aqueous extracts obtained using a modified short flax fiber; Daphnia magna Straus was used as a test object.

The assessment of water toxicity after contact with the obtained adsorption material was defined by the method of biotesting for Daphnia mortality. The acute toxicity of an aqueous extract after 24 h contact with the sorbent was calculated by the following formula:

(17) $$T=\frac{X_{w}-X_{t}}{X_{w}}\cdot 100\%,$$

where *T* is the percentage of Daphnia mortality in the experimental water, calculated in comparison with the control experiment; *Xt* is the number of surviving Daphnia in the control group; *X_w_* is the number of surviving Daphnia in the water.

Biotesting of the aqueous extract of the modified flax samples showed that Daphnia mortality after 48 h was 0%. Thereby, the obtained results indicate the complete absence of toxic effects in the case of a flax fiber sample modified with H-acid.

### 4.9. The Effect of Chemical Modification of Flax Fiber on the Sorption Properties of the Sorbent: The Mechanism of M^2+^ Sorption with Modified Sorbent

**Scheme 3:**
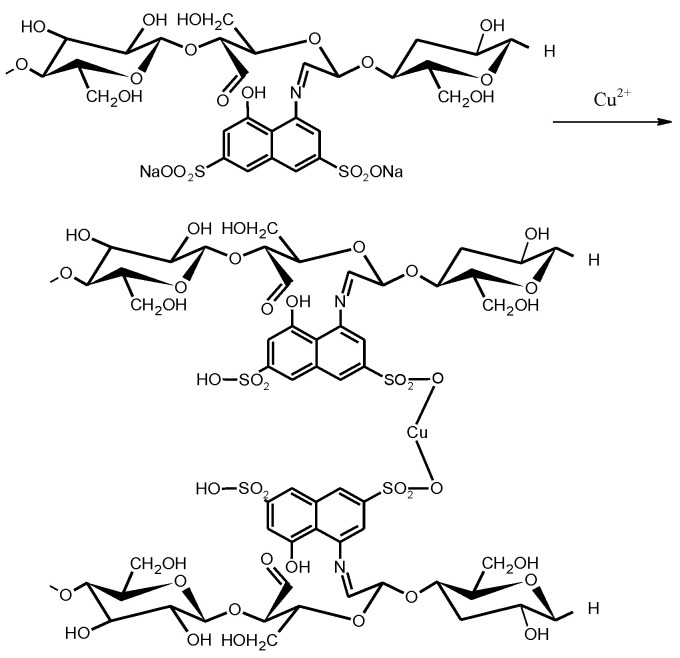
Sorption of Cu^2+^ ions by flax fiber modified with H-acid.

A suggested mechanism of metal ion sorption with modified flax fiber is shown in Scheme 3.

Hence, the sorbent capacity of the sorbent increases due to the fixation of strong acid sorption centers—sulfogroups at modification of flax with H-acid (the maximum sorption capacity of modified flax fibers is close to the capacity of an industrial cation exchanger).

## 5. Conclusions

The introduction of sorption-active groups determined the method for modifying cellulosic materials to produce biosorbents with a high sorption capacity (Cell–SO_3_H) and higher resistance to proton desorption in aqueous acidic media than Cell-COOH sorbents.

The kinetic curves of the heavy metal ion sorption by the sorbents under study are performed using pseudo first- and pseudo second-order kinetic models.

The heavy metal ion sorption of natural and modified flax fiber was best described with the pseudo second-order equation, and the theoretical *q_e_* values obtained were close to the experimental ones (*q_e exp_*) (correlation coefficient reached 0.99). It was found that the pseudo second-order model was more appropriate to the adsorption kinetics demonstrated by a high correlation coefficient.

The experimental sorption isotherms were correctly described using the Langmuir model (correlation coefficient of 0.99), and the limiting adsorption capacity decreased in the following sequence: Cu (II) > Cd (II) > Fe (II). The *A*_∞_ values for the modified sorbent increased by about 2.4–2.9 times compared with those for unmodified flax fiber.

The improvement in the sorption characteristics of flax fiber during the modification was caused by the appearance of new sorption-active sulfogroups in its structure. This was confirmed by the data from IR spectroscopy and elemental analysis.

SEM microscopic studies show changes in the surface structure and elemental composition of the flax fiber during its oxidation to dialdehyde cellulose and modification with H-acid.

## Figures and Tables

**Table 1:** Constants and correlation coefficients of the pseudo first- and the pseudo second-order kinetics for Cd^2+^, Cu^2+^, and Fe^2+^ ion sorption from aqueous solutions on short flax fiber.

Equilibrium Values			Pseudo First Order			Pseudo Second Order		
Metal Ion	*q_e exp_*, mg g^−1^	Removal Efficiency, %	*q_e_*, mg g^−1^	*k*_1_, min^−1^	*R* ^2^	*q_e_*, mg g^−1^	*k*_2_, mg min g^−1^	*R* ^2^
natural flax fiber:								
Cu^2+^	0.66	70.20	0.74	0.24	0.95	0.77	0.81	0.99
Cd^2+^	0.99	60.00	0.69	0.27	0.93	1.06	1.50	0.99
Fe^2+^	0.55	59.57	0.61	0.26	0.99	0.64	1.03	0.99
modified flax fiber:								
Cu^2+^	0.87	99.57	0.18	0.26	0.87	0.88	6.45	0.99
Cd^2+^	1.68	97.33	0.52	0.15	0.80	1.75	1.59	0.99
Fe^2+^	0.89	91.28	0.60	0.34	0.96	0.93	2.70	0.99

**Table 2:** Isotherm constants for Cu^2+^, Cd^2+^, and Fe^2+^ ion sorption by the flax fiber.

Langmuir Model			
Metal cation	*A*_∞_, mol kg^−1^	*K_L_*, L mol^−1^	*R* ^2^
Unmodified flax fiber			
Cu^2+^	0.68	1148.89	0.99
Cd^2+^	0.64	191.95	0.98
Fe^2+^	0.48	171.91	0.96
Modified flax fiber			
Cu^2+^	1.99	337.26	0.99
Cd^2+^	1.55	591.89	0.99
Fe^2+^	1.42	533.50	0.99
**Freundlich Model**			
Metal cation	*n*	*K_F_*, (mg g^−1^)/(L mg^−1^)^1/n^	*R* ^2^
Unmodified flax fiber			
Cu^2+^	2.19	4.84	0.89
Cd^2+^	1.79	4.75	0.98
Fe^2+^	2.33	1.73	0.95
Modified flax fiber			
Cu^2+^	2.32	9.31	0.89
Cd^2+^	1.83	13.76	0.94
Fe^2+^	1.87	11.34	0.95

**Table 3:** A comparison of maximum adsorption capacities on the different low-cost adsorbents on the base of flax.

Adsorbate	Biosorbent Based on the Following:	Maximum Adsorption	Adsorption Units	References
Cu	Unmodified flax fibers	0.68	mole·kg^−1^	Current study
Cd		0.64		
Fe		0.48		
Cu	Modified flax fibers	1.99	mole·kg^−1^	Current study
Cd		1.55		
Fe		1.42		
Cu	Flax processing waste	9.921	mg·g^−1^	[17]
Pb		10.741		
Zn		8.453		
Cu	Flax processing waste	8.3	mg·g^−1^	[18]
Pb		13.4		
Zn		7.1		
Cu	Flax tows	121.57	mmole·kg^−1^	[19]
Pb		111.68		
Zn		76.40		

## Data Availability

Detailed results of the author’s research are presented in the sources given in the References section.

## Supplementary Materials

The following supporting information can be downloaded at: https://www.mdpi.com/article/10.3390/polym15214212/s1. Figure S1: Pseudo first-order sorption kinetics related to Cd^2+^ (1, 3), Cu^2+^ (2, 6) and Fe^2+^ (4, 5) ions sorption on native (2, 3, 4) and modified (1, 5, 6) flax fiber; Figure S2: Pseudo second-order sorption kinetics related to Fe^2+^ (1, 4), Cu^2+^ (2, 3) and Cd^2+^ (5, 6) ions sorption on native (1, 2, 5) and modified (3, 4, 6) flax fiber; Figure S3: Intraparticle diffusion plot for Cd^2+^ (1, 2), Cu^2+^ (3, 5), and Fe^2+^ (4, 6) ions sorption on native (2, 5, 6) and modified (1, 3, 4) flax fiber from aqueous solutions; Figure S4: Linearization of sorption isotherms of Fe^2+^ (1, 4), Cd^2+^ (2, 5), and Cu^2+^ (3, 6) ions from aqueous solutions of their salts on native (1, 2, 3) and modified (4, 5, 6) flax fiber with Langmuir model; Figure S5: Linearization of sorption isotherms of Cu^2+^ (1, 4), Cd^2+^ (2, 5), and Fe^2+^ (3, 6) ions from aqueous solutions of their salts on modified (1, 2, 3) and native (4, 5, 6) flax fiber with Freundlich model; Figure S6: Elemental analysis of flax fiber samples: a—native flax fibers; b—flax fibers oxidized with sodium metaperiodate; c—flax fibers modified with AHD-acid; Figure S7: Elemental analysis of flax fiber samples after sorption of copper (II) ions: *a*—native flax fiber; *b*—oxidized flax fiber; *c*—flax fiber modified with AHD-acid; Table S1: The parameters of the Weber-Morris intra-particle diffusion model.
