# Supplementary material for: Heavy Metal Ions(II) Sorption by a Cellulose-Based Sorbent Containing Sulfogroups
Source: Polymers (Basel). 2023 Oct 24;15(21):4212. doi: 10.3390/polym15214212 (PMC10649064; doi:10.3390/polym15214212)
Supplement: Supplementary file 1 [file polymers-15-04212-s001.zip › polymers-2576676-supplementary.docx]

Supplementary Materials

Supplementary Material S1

Processing of kinetic curves of heavy metal ions sorption by native and modified flax fibers in the framework of pseudo first- and pseudo second-order kinetic models is shown in Figures S1 and S2.

**Figure S1.** Pseudo first-order sorption kinetics related to Cd^2+^ (1, 3), Cu^2+^ (2, 6) and Fe^2+^ (4, 5) ions sorption on native (2, 3, 4) and modified (1, 5, 6) flax fiber.

**Figure S2.** Pseudo second-order sorption kinetics related to Fe^2+^ (1, 4), Cu^2+^ (2, 3) and Cd^2+^ (5, 6) ions sorption on native (1, 2, 5) and modified (3, 4, 6) flax fiber.

Supplementary Material S2

**Figure S3.** Intraparticle diffusion plot for Cd^2+^ (1, 2), Cu^2+^ (3, 5), and Fe^2+^ (4, 6) ions sorption on native (2, 5, 6) and modified (1, 3, 4) flax fiber from aqueous solutions.

The plots on Figure S3 include three linear segments having different slopes which indicates that the process takes place in three stages and its mechanism is not determined only by the intraparticle diffusion.

Step 1: film diffusion (the metal ions move from the solution to the external surface of the adsorbent through film diffusion);

Step 2: intraparticle diffusion (the solute ions transfer from the exterior surface to the interior pores of the adsorbent through the intraparticle diffusion mechanism);

Step 3: equilibrium stage (intraparticle diffusion remains constant due to the pore volume is exhausted).

Table S1 contains the parameters of the Weber-Morris intraparticle diffusion model for the sorption of Cd^2+^, Cu^2+^, and Fe^2+^ ions on native and modified flax fiber determined by using the equation (7). The values of C_i_ in this table corresponds to the thickness of the boundary layer.

**Table S1.** The parameters of the Weber-Morris intra-particle diffusion model.

| **Ion** | **Step** | **K_id_, mg·g^-1^ min^-1/2^** | **C_i_, mg·g^-1^** | **R^2^** |
| --- | --- | --- | --- | --- |
| Unmodified flax fiber | | | | |
| Cu^2+^  Cd^2+^  Fe^2+^ | 1  2  3  1  2  3  1  2  3 | 0.293  0.198  0.026  0.436  0.197  0.015  0.267  0.218  0.026 | 0.028  0.183  0.578  0.208  0.527  0.942  0.021  0.036  0.381 | 0.99  0.99  0.89  0.97  0.99  0.93  0.98  0.97  0.99 |
| Modified flax fiber | | | | |
| Cu^2+^  Cd^2+^  Fe^2+^ | 1  2  3  1  2  3  1  2  3 | 0.366  0.217  0.025  0.489  0.159  0.078  0.412  0.215  0.041 | 0.184  0.441  0.819  0.744  1.228  1.433  0.178  0.354  0.788 | 0.99  0.99  0.89  0.99  0.98  0.93  0.98  0.93  0.99 |

As shown in Figure S3 and Table S1, the linear segments did not pass through the origin, indicating that the adsorption process involved the intraparticle diffusion mechanism, but this was not the only rate-controlling step.

Thus, chemisorption plays a major role in the sorption process as a whole, while film diffusion and intraparticle diffusion control the early stage of the sorption process.

Supplementary Material S3

The obtained experimental data were processed using the Langmuir and Freundlich models (Figures S4 and S5).

**Figure S4.** Linearization of sorption isotherms of Fe^2+^ (1, 4), Cd^2+^ (2, 5), and Cu^2+^ (3, 6) ions from aqueous solutions of their salts on native (1, 2, 3) and modified (4, 5, 6) flax fiber with Langmuir model.

**Figure S5.** Linearization of sorption isotherms of Cu^2+^ (1, 4), Cd^2+^ (2, 5), and Fe^2+^ (3, 6) ions from aqueous solutions of their salts on modified (1, 2, 3) and native (4, 5, 6) flax fiber with Freundlich model.

Supplementary Material S4

Elemental analysis of flax fiber samples before (Figure S6) after sorption of copper (II) compounds (Figure S7).

| 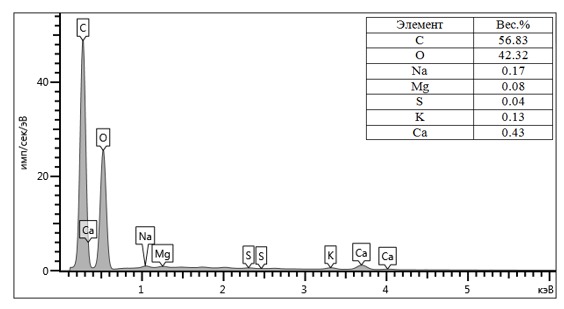 | 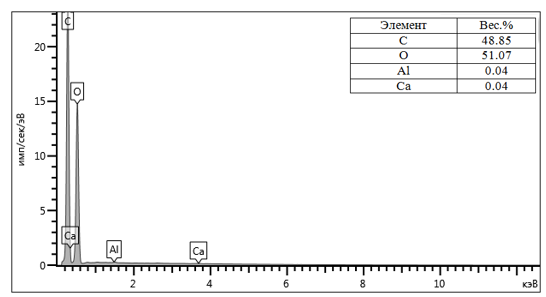 |
| --- | --- |
| (**a**) | (**b**) |
| 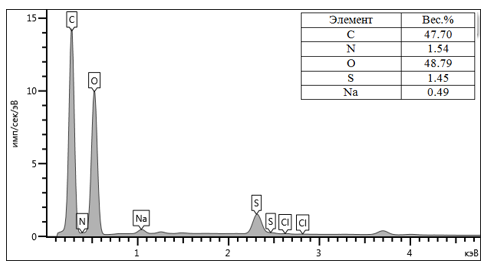 |  |
| (**c**) |  |

**Figure S6.** Elemental analysis of flax fiber samples: a—native flax fibers; b—flax fibers oxidized with sodium metaperiodate; с—flax fibers modified with AHD-acid.

The data presented in Figure 4.1 show that the elemental composition of the native flax fiber changed as the result of its oxidation and modification. In the oxidized sample, the oxygen content increases from 42% to 51%, compared with the pristine one, and sulfur (1.45%) and nitrogen (1.54%) appear in the elemental composition of the modified sample, which confirms the incorporation of AHD-acid into the sorbent structure.

The data presented in Figure 4.2 shows that the elemental composition of the modified flax fiber after the sorption of Cu^2+^ ions was essentially changed.

| 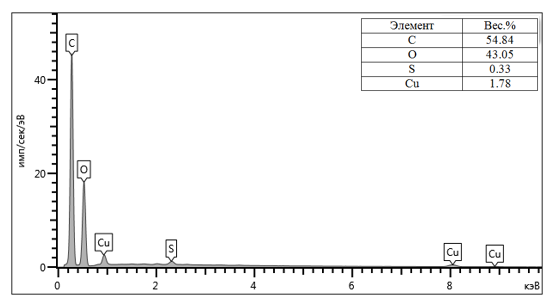 | 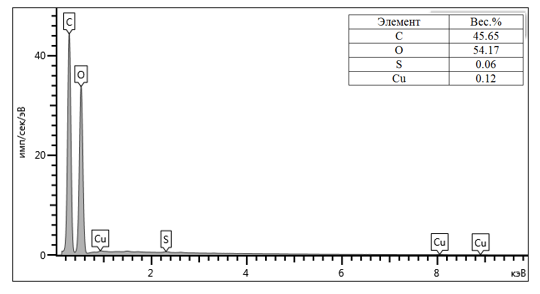 |
| --- | --- |
| (**a**) | (**b**) |
| 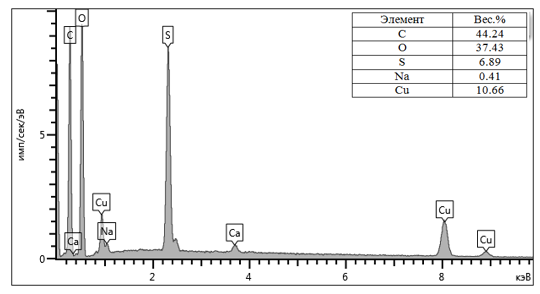 |  |
| (**c**) |  |

**Figure S7.** Elemental analysis of flax fiber samples after sorption of copper (II) ions: *a*—native flax fiber; *b—*oxidized flax fiber; *c—*flax fiber modified with AHD-acid.
